# Analysis of COVID-19 Infection and Mortality Among Patients With Psychiatric Disorders, 2020

**DOI:** 10.1001/jamanetworkopen.2021.34969

**Published:** 2021-11-23

**Authors:** Antonio L. Teixeira, Trudy Millard Krause, Lopita Ghosh, Lokesh Shahani, Rodrigo Machado-Vieira, Scott D. Lane, Eric Boerwinkle, Jair C. Soares

**Affiliations:** 1 Department of Psychiatry and Behavioral Sciences, McGovern Medical School, University of Texas Health Science Center at Houston, Houston; 2 School of Public Health, The University of Texas Health Science Center at Houston, Houston

## Abstract

**Importance:**

People with major psychiatric disorders are more likely to have comorbidities associated with worse outcomes of COVID-19. This fact alone could determine greater vulnerability of people with major psychiatric disorders to COVID-19.

**Objective:**

To assess the odds of testing positive for and mortality from COVID-19 among and between patients with schizophrenia, mood disorders, anxiety disorders and a reference group in a large national database.

**Design, Setting, and Participants:**

This cross-sectional study used an electronic health record data set aggregated from many national sources in the United States and licensed from Optum with current and historical data on patients tested for COVID-19 in 2020. Three psychiatric cohorts (patients with schizophrenia, mood disorders, or anxiety disorders) were compared with a reference group with no major psychiatric conditions. Statistical analysis was performed from March to April 2021.

**Exposure:**

The exposures observed include lab-confirmed positivity for COVID-19 and mortality.

**Main Outcomes and Measures:**

The odds of testing positive for COVID-19 in 2020 and the odds of death from COVID-19 were measured.

**Results:**

The population studied included 2 535 098 unique persons, 3350 with schizophrenia, 26 610 with mood disorders, and 18 550 with anxiety disorders. The mean (SD) age was 44 (23) years; 233 519 were non-Hispanic African American, 1 583 440 were non-Hispanic Caucasian; and 1 580 703 (62%) were female. The schizophrenia cohort (positivity rate: 9.86%; adjusted OR, 0.90 [95% CI, 0.84-0.97]) and the mood disorder cohort (positivity rate: 9.86%; adjusted OR, 0.93 [95% CI, 0.87-0.99]) had a significantly lower rate of positivity than the anxiety disorder cohort (positivity rate: 11.17%; adjusted OR, 1.05 [95% CI, 0.98-1.12) which was closer to the reference group (11.91%). After fully adjusting for demographic factors and comorbid conditions, patients with schizophrenia were nearly 4 times more likely to die from the disease than the reference group (OR, 3.74; 95% CI, 2.66-5.24). The mood disorders COVID-19 cohort had a 2.76 times greater odds of mortality than the reference group (OR, 2.76; 95% CI, 2.00-3.81), and the anxiety disorders cohort had a 2.39 times greater odds of mortality than the reference group (OR, 2.39; 95% CI, 1.68-3.27).

**Conclusions and Relevance:**

By examining a large database while controlling for multiple confounding factors such as age, race and ethnicity, and comorbid medical conditions, the present study found that patients with schizophrenia had much increased odds of mortality by COVID-19.

## Key Points

**Question:**

Do people with major psychiatric disorders have greater odds of testing positive for COVID-19 and dying from it?

**Findings:**

This cross-sectional study based on nationwide electronic health record data from 2 535 098 participants showed that the schizophrenia and mood disorder cohorts had significantly lower rates of positivity (9.86%) than the anxiety disorder cohort (11.17%) and the general population (11.91%). Conversely, patients with schizophrenia had a higher rate of death from COVID-19 than the reference group or those with mood disorders or anxiety disorders.

**Meaning:**

This study suggests that patients with major psychiatric disorders may be more likely to have medical comorbidities associated with worse COVID-19 outcome and yet have a higher mortality rate independent of comorbidities.

## Teaser

This cross-sectional study examines the odds of COVID-19 infection and mortality among US patients with schizophrenia, mood disorders, anxiety disorders compared with a reference group.

## Introduction

In March 2020, the spread of SARS-CoV-2 was declared a world pandemic by the World Health Organization. As of March 23, 2021, more than 122 million cases of SARS-CoV-2 infection and more than 2.7 million COVID-19–related deaths have been reported worldwide.^1^ SARS-CoV-2 primarily affects the respiratory system, and although the great majority of infected individuals are asymptomatic or have mild symptoms, approximately 15% experience severe disease, and approximately 5% will develop a life-threatening illness.^2^ Besides the respiratory system, SARS-CoV-2 can affect virtually all organs.^3,4^ A growing body of evidence suggests that SARS-CoV-2 infection can affect the central nervous system (CNS), either directly or indirectly, and is associated with multiple neurological symptoms, from olfactory and gustatory dysfunction to more severe and uncommon events such as Guillain-Barré syndrome and encephalitis.^5,6,7,8,9^

Great attention has also been dedicated to acute and chronic psychiatric symptoms associated with COVID-19.^10,11,12^ In a prospective cohort study, anxiety, depression, and posttraumatic symptoms were observed in 23%, 18%, and 7%, respectively, of survivors of COVID-19 who had been hospitalized in an intensive care unit 4 months earlier.^13^ Patients frequently report persistent fatigue, sleep, and cognitive problems, the latter commonly referred to as brain fog.^14^ In addition, emerging evidence shows that patients with premorbid psychiatric disorders are at increased risk of SARS-CoV-2 infection^15,16,17^ and more severe outcomes of COVID-19.^17,18,19^ In a pivotal study, Nemani et al^20^ reported that the diagnosis of a schizophrenia spectrum disorder, but not mood or anxiety disorders, was a risk factor for mortality in patients with COVID-19 behind only age in strength of association. However, this study had limitations, including its definition of mortality (ie, death or discharge to hospice), sample from a single health system, relatively small number of patients in each psychiatric diagnostic category, and limited control of potential confounders due to available data (eg, metabolic syndrome, obesity, body mass index, antipsychotic use).

The Optum COVID-19 Electronic Health Record (EHR) database contained 3 614 884 patient records derived from a network of health care provider organizations across the United States, providing a unique opportunity to evaluate the affect of COVID-19 on patients with psychiatric disorders at a nationally representative level. Leveraging the access to this national COVID-19 EHR database, we aimed to explore whether people with major psychiatric disorders had higher rates of infection and mortality associated with COVID-19. Given that people with major psychiatric disorders have several factors associated with worse COVID-19 outcomes, we hypothesized they were more vulnerable to the infection.

## Methods

### Study Design

Data from the national COVID-19 EHR database was certified as de-identified by an independent statistical expert following HIPAA statistical de-identification rules and managed according to Optum® customer data use agreements. Clinical and administrative data were linked by Optum® before de-identification. The COVID-19 data incorporates clinical and medical administrative data from both inpatient and ambulatory electronic health records (EHRs), practice management and other internal systems. Information is processed from across the continuum of care, including acute inpatient stays and outpatient visits. The COVID-19 data capture point-of-care information specific to the patient with COVID-19 during initial presentation, acute illness, and convalescence with over 500 mapped laboratory tests and bedside observations.

The study was approved by the University of Texas Health Science Center at Houston institutional review board with a waiver of authorization for informed consent based on exempt status according to 45 CFR 46.101(b) and the deidentification of participants and its retrospective and observational nature. This study adhered to the Strengthening the Reporting of Observational Studies in Epidemiology (STROBE) reporting guideline.

### Population and Definitions

The data set comprised a cohort of individuals who received a laboratory test (coded by Logical Observation Identifiers Names and Codes [LOINC] or *Current Procedural Terminology [CPT]* codes or identified by test name in the EHR text) for COVID-19 between February and December 2020. Once included in the cohort, all available EHRs from contributing sources were included as far back as 2010, which enabled identification of medical and psychiatric comorbidities.

There was a total of 3 614 884 participants in the cohort, with the total test count at 7 318 988, reflective of multiple tests per participant across the year. Nearly 24% of the cohort were excluded due to being aged less than 18 years, nondefinitive test results, or missing historical data (ie, the only record was of the COVID-19 test), thus there were a total of 2 769 074 participants available for data analyses (eFigure in the Supplement). Confirmation of COVID-19 was derived by documentation of a positive polymerase chain reaction result. A positivity rate of 11.4% yielded 317 849 patients who tested positive for COVID-19 and 2 451 225 patients who tested negative.

We extracted *International Statistical Classification of Diseases* psychiatric diagnostic codes using *International Classification of Diseases, Ninth Revision (ICD-9)*, *International Statistical Classification of Diseases and Related Health Problems, Tenth Revision (ICD-10)*, and Systematized Nomenclature of Medicine–Clinical Terms (SNOMED CT) diagnoses codes from the EHR. Patients were categorized in a hierarchical fashion into 3 mutually exclusive psychiatric diagnostic categories based on codes documented before March 1, 2020: (1) schizophrenia spectrum disorders, (2) mood disorders, and (3) anxiety disorders (eTable in the Supplement). Patients were compared with a reference group of all remaining participants with laboratory tests for COVID-19 in 2020. Of note, there was a subset of patients taking psychotropic drugs, but without a psychiatric diagnosis in the EHR. This group (n = 233 936) had sociodemographic and clinical parameters very similar to the reference group. To control for possible confounding due to exposure to psychotropic medication between the 3 psychiatric groups and the reference group, patients prescribed psychotropic medications but without documented psychiatric diagnoses were excluded from the formal data analyses. Thus, the final sample included 2 535 098 patients, of which 294 417 were COVID-19 positive (approximately 12%) (eFigure in the Supplement). Each of the 4 cohorts was then divided into COVID-19 positive vs negative cases.

### Covariates and Confounders

The following variables were collected: age, gender, race and ethnicity, geographic region, body mass index, and smoking status, along with medical comorbidities: hypertension, diabetes, chronic kidney disease, coronary artery disease, ischemic heart disease, metabolic syndrome and chronic obstructive pulmonary disease. Race and ethnicity were determined by patient report and were included due to established differences in COVID-19–related mortality by race.^21^ Gender was also self-reported. Medical comorbidities were extracted from encounters before March 1, 2020, and included diagnoses documented from the medical history, and inpatient and outpatient problem list. We identified different features of patients who were either COVID-19 positive and negative for each of the 3 defined psychiatric diagnostic groups and the reference group to assess mortality odds.

### Outcome Measures

The prevalence of psychiatric disorders among all patients tested for COVID-19 was first determined to identify the 4 cohorts: (1) schizophrenia, (2) mood disorders, (3) anxiety disorders, and (4) reference group.

The outcomes of comparison were likelihood of testing positive for COVID-19 and COVID-19–related death in 2020. The primary outcome measure was risk of death from COVID-19 across the 3 psychiatric diagnostic groups and the reference group. First, descriptive statistics were used to report the likelihood of a positive test result in each psychiatric diagnostic group compared with the reference group for an initial outcome measure. Within COVID-19–positive cases, mortality outcomes were compared between each psychiatric diagnostic group and the reference group using adjusted odds ratios (aORs). The primary outcome of death is widely used when assessing risk related to COVID-19 infection and is readily identified in the data by the date of death field and treated as a binary variable. Death was linked to the COVID-19 condition when it occurred during a hospitalization for COVID-19 or if it occurred within 7 days of treatment for COVID-19.

### Statistical Analysis

The statistical results were generated using SAS software version 9.4 (SAS Institute Inc) from Month Year to Month Year. Mortality outcome was compared between each psychiatric diagnostic group and the reference group using odds ratios (ORs). Three types of ORs were estimated: (1) unadjusted; (2) demographically adjusted for gender, age, race and ethnicity; and (3) fully adjusted for the statistically significant demographic factors plus chronic medical conditions and smoking status. Logistic regression models that included indicators for the psychiatric diagnostic categories (3 indicators with the control group as reference) were used to estimate ORs and the covariates were included to estimate the fully adjusted ORs; 95% CIs were estimated based on those models. Using logistic regression, we determined significance with a 2-sided test, setting *P* < .05 as the significance threshold.

## Results

The population studied included 2 535 098 unique persons, 3350 with schizophrenia, 26 610 mood disorders, 18 550 anxiety disorders. The mean (SD) age was 44 (23) years; 233 519 were non-Hispanic African American, 1 583 440 were non-Hispanic Caucasian; and 1 580 703 (62%) were female. Table 1 provides descriptive details of the cohorts. Schizophrenia was diagnosed in 1.23% of the participants (n = 33 960), mood disorders in approximately 10% (n = 269 844), and anxiety disorders in 6% (n = 116 350). All variables reviewed, with the exception of age, ischemic heart disease and metabolic syndrome, showed significant differences between the study cohorts of psychiatric diagnoses compared with the reference group without the psychiatric diagnoses.

**Table 1.** Baseline Characteristics of Participants With Positive and Negative SARS-CoV-2 Test Results According to Psychiatric Diagnostic Categories

Characteristic	Participants, No. (%)								
	Total (N = 2 535 098)	Schizophrenia (1.23% of population)		Mood disorders (9.74% of population)		Anxiety disorders (6.10% of population)		Reference group (74.48% of population)	
		COVID^+^ (n = 3350)	COVID^−^ (n = 30 610)	COVID^+^ (n = 26 610)	COVID^−^ (n = 243 234)	COVID^+^ (n = 18 856)	COVID^−^ (n = 149 991)	COVID^+^ (n = 245 601)	COVID^−^ (n = 1 816 846)
% Of total	NA	0.12	1.11	0.96	8.78	0.68	5.42	8.87	65.61
Positivity rate, %	11.61	9.86	NA	9.86	NA	11.17	NA	11.91	NA
Gender									
Female	1 580 703 (62)	1845 (55)	16 739 (55)	19 221 (72)	175 956 (72)	12 900 (68)	103 450 (69)	124 403 (51)	968 642 (53)
Male	1 185 282 (38)	1504 (45)	13 850 (45)	7375 (28)	67 083 (28)	5944 (32)	46 460 (31)	120 929 (49)	845 841 (47)
Age, mean (SD), y	44 (22)	57 (19)	54 (18)	50 (19)	50 (18)	48 (18)	50 (19)	43 (21)	44 (23)
Race and ethnicity, No.									
African American Hispanic	6412	10	102	107	526	50	292	959	4366
African American not Hispanic	233 519	609	5339	2217	17 730	1371	8834	28 927	168 492
African American unknown	20 404	38	269	108	1072	50	534	2308	16 025
Asian Hispanic	790	1	3	9	43	5	33	122	574
Asian not Hispanic	45 689	29	261	168	1385	119	1147	4844	37 736
Asian unknown	5105	4	13	11	118	15	76	479	4389
Caucasian Hispanic	72 454	133	975	926	5414	640	3249	11 762	49 355
Caucasian not Hispanic	1 583 440	2173	20 452	19 897	188 432	14 562	119 509	130 901	1 087 514
Caucasian unknown	157 802	115	1362	900	13 694	571	7475	11 955	121 730
Other or unknown^a^	105 805	238	3289	2267	14 820	2980	10 017	53 344	326 665
% Caucasian	72	72	74	82	85	84	87	63	69
Current smoker	389 120 (15)	800 (24)	11 890 (39)	3897 (15)	62 111 (26)	2285 (12)	30 364 (20)	17 908 (7)	216 635 (12)
Chronic health conditions									
Hypertension	427 328 (17)	1337 (40)	10 784 (35)	5942 (22)	59 535 (24)	3416 (18)	32 329 (22)	29 252 (12)	230 156 (13)
Diabetes	203 400 (8)	856 (40)	6340 (21)	3509 (13)	31 624 (13)	1687 (9)	14 437 (10)	15 912 (6)	101 092 (5)
BMI ≥25	1 099 937 (43)	1208 (36)	12 069 (39)	11 179 (42)	109 406 (45)	7272 (39)	61 151 (41)	98 240 (40)	799 412 (44)
COPD	125 116 (5)	623 (18)	5651 (18)	2141 (8)	24 780 (10)	1014 (5)	11 952 (8)	5331 (2)	54 311 (0.3)
Ischemic heart disease	445 844 (18)	1638 (49)	13 644 (45)	6696 (25)	63 908 (26)	4007 (21)	34 997 (23)	27 807 (11)	233 650 (13)
Metabolic syndrome	32 825 (1)	129 (4)	1043 (3)	892 (3)	7537 (3)	447 (2)	3297 (2)	1956 (0.7)	12 588 (0.7)
Current psychiatric medications									
Antidepressants	NA	1216 (36)	11 937 (39)	17 859 (67)	166 939 (69)	8332 (44)	67 639 (45)	0	0
Antipsychotics	NA	1065 (32)	10 489 (34)	3314 (12)	38 003 (16)	641 (3)	6769 (4)	0	0
Tested at an inpatient facility									
Inpatient	NA	873 (26)	7622 (25)	3219 (12)	36 585 (15)	1545 (8)	16 643 (11)	26 693 (11)	214 748 (12)
Inpatient psychiatric facility	NA	107 (3)	2025 (7)	191 (0.7)	4209 (2)	49 (0.2)	873 (0.5)	531 (0.2)	5909 (0.3)
Died in 2020 post COVID for COVID + or died in 2020 for COVID −	42 153	287	1075	910	4915	454	2431	5899	26 182
Rate of death, %	1.66	8.57	3.51	3.42	2.02	2.41	1.62	2.40	1.44

Abbreviations: BMI, body mass index (calculated as weight in kilograms divided by height in meters squared); COPD, chronic obstructive pulmonary disease; COVID −, tested negative for COVID-19; COVID +, tested positive for COVID-19; NA, not applicable.

^a^ Data provided race as African American, Asian, Caucasian, other/unknown. Ethnicity was Hispanic, Not Hispanic, and unknown.

The psychiatric cohorts had a higher proportion of female participants than the reference group, although the population with schizophrenia was more closely aligned with the reference group than the mood disorders or anxiety disorders cohorts. The race and ethnicity was also different, with 68% Caucasian in the reference group and between 72% and 87% Caucasian in the psychiatric diagnosis groups. Rates of all chronic comorbidities were also greater in the psychiatric cohorts than the reference group. Obesity rates for schizophrenia were between 8% to 15% lower than the comparison groups. Consistent with established estimates, current smoking status was higher for all psychiatric cohorts than the reference group. Variables that were statistically significantly different between the psychiatric groups and the reference group were used to adjust the ORs for COVID-19 positivity and mortality.

Table 2 provides the adjusted ORs and 95% CIs for fully adjusted comparisons with the reference group for the likelihood of testing positive for COVID-19. The fully adjusted models for testing positive for COVID-19 and for mortality dropped 2 comorbid conditions (ischemic heart disease and metabolic syndrome) for lack of significance.

**Table 2.** Adjusted Odds Ratio of Testing Positive for COVID-19

Characteristic	Adjusted OR (95% CI)
Schizophrenic vs reference	0.90 (0.84-0.97)
Mood disorders vs reference	0.93 (0.87-0.99)
Anxiety disorders vs reference	1.05 (0.98-1.12)
Age	1.00 (1.00-1.00)
Gender: female vs male	0.89 (0.88-0.89)
Race^a^	
African American vs Caucasian	1.37 (1.36-1.39)
Asian vs Caucasian	1.01 (0.98-1.04)
Other or unknown vs Caucasian	1.25 (1.24-1.27)
Smoke	1.73 (1.71-1.76)
Ischemic heart disease	0.81 (0.79-0.82)
Hypertension	0.70 (0.69-0.70)
Diabetes	1.03 (1.01-1.05)
BMI ≥25	0.87 (0.84-0.90)

Abbreviations: BMI, body mass index (calculated as weight in kilograms divided by height in meters squared); OR, odds ratio.

^a^ Data provided race as African American, Asian, Caucasian, other/unknown.

Regarding SARS-CoV-2 infection, the overall positivity rate was 11.91% for the general population and in the reference group. Female participants were less likely to test positive, as were smokers and most comorbid conditions with the exception of diabetes, which increased the odds by nearly 18%. The positivity rate for the schizophrenia population (9.86%; adjusted OR, 0.90 [95% CI, 0.84-0.97]) matched that of the mood disorders cohort (9.86%; adjusted OR, 0.93 [95% CI, 0.87-0.99]), which were the lowest, with the anxiety disorders cohort at 11.17% (OR, 1.05 [95% CI, 0.98-1.12]). In all groups, the West North Central region had the highest positivity rate, with a high of 18% (62 572 of 347 802) for the reference group. Interestingly, although only 12% of the reference group (n = 247 881) received their laboratory test for COVID-19 at an inpatient facility, 31% of the schizophrenia group (n = 10 627) (vs 16% for mood disorders [n = 44 204] and 10% for anxiety disorders [n = 19 110]) received their positive test at an inpatient facility.

In line with a greater burden of medical comorbidities and smoking status (Table 1), despite the lower positivity rate, the schizophrenia cohort had the highest rate of mortality. More than 8% of the patients with schizophrenia who tested positive for COVID-19 (n = 287) died compared with 2% of the reference group (n = 5899), and 3.5% of the patients with schizophrenia who tested negative for COVID-19 (n = 1075) died during the pandemic year compared with 1.4% of the reference group (n = 2431). Table 3 provides the adjusted ORs and 95% CIs of the significant variables on the likelihood of death from COVID-19 for each positive study group. Ethnicity, chronic obstructive pulmonary disease, and metabolic syndrome were not statistically associated with mortality. Most chronic health conditions increased the odds of mortality with the exception of obesity, with hypertension increasing the odds nearly 80% (OR, 1.79; 95% CI, 1.53-2.19). The concordance of a model with all significant variables was 0.89, which indicates an 89% chance of accurately indicating mortality.

**Table 3.** Adjusted Odds Ratio of Mortality for COVID-19 Positive Psychiatric Cohorts

Characteristic	Adjusted OR (95% CI)
Schizophrenic vs reference	3.74 (2.66-5.24)
Mood disorders vs reference	2.76 (2.00-3.81)
Anxiety disorders vs reference	2.34 (1.68-3.27)
Age	1.10 (1.10-1.11)
Gender female vs male	0.61 (0.58-0.64)
Race^a^	
African American vs Caucasian	1.82 (1.70-1.94)
Asian vs Caucasian	1.69 (1.43-2.01)
Other or unknown vs Caucasian	1.49 (1.36-1.63)
Smoke	1.26 (1.20-1.32)
Ischemic heart disease	1.53 (1.36-1.73)
Hypertension	1.79 (1.53-2.09)
Diabetes	1.43 (1.23-1.66)
BMI ≥25	0.67 (0.64-0.70)

Abbreviations: BMI, body mass index (calculated as weight in kilograms divided by height in meters squared); OR, odds ratio.

^a^ Data provided race as African American, Asian, Caucasian, other/unknown.

The COVID-19–positive cohort with schizophrenia was nearly 4 times more likely to die from COVID-19 than the reference group (OR, 3.74; 95% CI, 2.66-5.24). The COVID-19 positive cohort with mood disorders had a 2.76 times greater odds of mortality than the reference group (OR, 2.76; 95% CI, 2.00-3.81), and the anxiety disorders cohort had a 2.39 times greater odds of mortality than the reference group (OR, 2.39; 95% CI, 1.68-3.27).

Table 4 provides the adjusted ORs comparing each of the psychiatric cohorts on the likelihood of testing positive for COVID-19. The schizophrenia group was 7% less likely to test positive than the reference group (OR, 0.93; 95% CI, 0.83-0.97). Table 4 further provides the ORs comparing the schizophrenia cohort with the other cohorts for mortality when positive for COVID-19. It found that the only statistically significant difference was between the schizophrenia cohort and the anxiety disorders cohort (OR, 1.60; 95% CI, 1.34-1.90).

**Table 4.** Adjusted Odds of Testing Positive for COVID-19 and Adjusted Odds of Mortality Compared Among Psychiatric Cohorts

Outcome and cohort comparison	OR (95% CI)	*P* value
Testing positive		
Schizophrenia vs mood disorders	0.90 (0.84-0.97)	<.001
Schizophrenia vs anxiety disorders	0.93 (0.87-0.99)	<.001
Mood disorders vs anxiety disorders	1.05 (0.98-1.12)	<.001
Mortality		
Schizophrenia vs mood disorders	1.35 (1.15-1.58)	<.001
Schizophrenia vs anxiety disorders	1.60 (1.34-1.90)	<.001
Mood disorders vs anxiety disorders	1.18 (1.04-1.34)	.01

Abbreviation: OR, odds ratio.

## Discussion

The main findings of this study were: (1) patients with schizophrenia and mood disorders displayed lower rates of infection with SARS-CoV-2; (2) patients with schizophrenia were more likely to be diagnosed with SARS-CoV-2 infection in an inpatient facility; (3) patients with schizophrenia had higher death rates due to COVID-19.

Although previous studies reported that patients with premorbid psychiatric disorders are at increased risk of SARS-CoV-2 infection,^15,16,17^ our data did not confirm this, actually showing the opposite. Using a large North American database of EHR, Wang et al^17^ found that participants with a recent (ie, within past year) diagnosis of a mental disorder had significantly higher rates of SARS-CoV-2 infection, an effect strongest for depression (OR, 7.64; 95% CI, 7.45-7.83; *P* < .001) and schizophrenia (OR, 7.34; 95% CI, 6.65-8.10; *P* < .001).^17^ The OR for infection was further increased among African Americans and women. A similar trend was observed in participants with a lifetime diagnosis of a mental disorder, but the associations were much lower (eg, for depression: OR, 2.01; 95% CI, 1.96-2.06, *P* < .001; for schizophrenia: OR, 1.48; 95% CI, 1.33-1.65; *P* < .001). However, the authors focused their analyses only on patients with a recent diagnosis. Moreover, although the sample was large (approximately 1.3 participants with recent mental disorder), the rate of infection was very low, with only 3430 reported cases of COVID-19 among this population. Compared with our infection numbers (>290 000), these results might not necessarily reflect the susceptibility of these individuals to the dynamic landscape of the infection. Taquet et al^15^ also reported that having a psychiatric disorder in the year before the COVID-19 outbreak was associated with increased risk of COVID-19 (relative risk, 1.65; 95% CI, 1.59-1.71; *P* < .001). This result was not related to any specific psychiatric diagnosis, and was similar regardless of whether the diagnosis was made within 1 or 3 years of the outbreak.^15^ It is worth noticing that the observed risk by Taquet et al^15^ was much smaller than previously reported.^17^ Finally, a Korean study did not find an association between psychiatric diagnosis and risk of COVID-19 diagnosis.^16^ These discrepant results might reflect not only methodological issues (eg, source and size of the sample) but also the stage of the outbreak. The 2 aforementioned studies showing the association between psychiatric disorder and risk of infection had very low positivity rate (<1%), whereas the Korean study (approximately 3%) and the current analysis (>10%) report much higher rates.

It is possible that patients with major psychiatric disorders—schizophrenia and mood disorders—being more socially withdrawn are less exposed to the virus, explaining the lower positivity rate in the current study. Alternatively, the lower positivity rate would reflect not a true reduced risk of infection, but lower testing numbers. Upon review of the site of COVID-19 testing, it was revealed that the patients with schizophrenia had a much higher rate (almost 40%) of receiving a test at an inpatient facility, including a psychiatric facility. These patients would ostensibly be less likely to seek testing in outpatient clinics or dedicated sites, probably undergoing COVID-19 tests when presenting related symptoms.

Patients with major psychiatric disorders are more likely to have medical comorbidities associated with worse COVID-19 outcomes.^22,23^ Our results found that especially patients with schizophrenia had higher frequency of medical comorbidities. Compared with the reference group and the other psychiatric cohorts, patients with schizophrenia had a much higher rate of death from COVID-19. Importantly, patients with schizophrenia had an increased mortality, even after adjusting for chronic medical comorbidities and smoking. Moreover, our results were quite similar to the ones reported by Nemani et al^20^ in a much smaller sample. Results of the current study also suggest that patients with major psychiatric disorders have a higher mortality that is independent of their medical comorbidities. Although the explanations for this finding remain elusive, it is tempting to speculate that an increased inflammatory response, a biological factor common to both severe COVID-19 pathophysiology (cytokine storm)^24,25^ and schizophrenia (chronic low grade inflammation),^26^ could be one of the mechanisms underlying this increased mortality.

### Limitations and Strengths

This study had some limitations. It may be limited by the design of the data set, which is aggregated from data by sources who voluntarily contribute EHR records. Not all clinicians from whom a patient seeks or has historically sought treatment may contribute, thus creating a potential for missing records. Furthermore, the data set is limited to individuals who tested for COVID-19 during 2020, thus creating a potential bias toward individuals with suspected symptoms or those otherwise seeking contact with health professionals. Another limitation may be the temporal element of the spread of COVID-19 across the United States regionally from the east coast to the west coast, along with the temporal effect of treatment practices being improved as the pandemic spread. Finally, the results are reflective of the experience within the United States and may not be replicated in other countries. Despite such limitations, the greatest strength of this cross-sectional study lies in the large sample size. Additional strengths include the focus on different patient groups and the adjustment for important confounders in the analyses such as demographic characteristics, comorbidities, obesity, and smoking.

## Conclusions

Results of the current study suggest that many patients with major psychiatric disorders demonstrate a lower rate of positive COVID-19 test during the 2020 pandemic than the general population. These rates may be influenced by a more limited exposure to SARS-CoV-2 due to social isolation. This study also found that the patients with major psychiatric disorders are more likely to have comorbidities associated with worse COVID-19 outcomes. Finally, patients with schizophrenia had higher mortality rates for COVID-19, independent of those comorbidities. This study’s findings suggest the need to foster recognition of pandemic risks on specific groups of patients with psychiatric conditions, and may drive alternative approaches to COVID-19 disease testing and interventions to improve clinical outcomes.

## Notes

Supplement. **eTable.** ICD-10 Codes Used to Define Each Cohort and Logic Steps **eFigure.** Flowchart
