# Supplementary material for: Analysis of COVID-19 Infection and Mortality Among Patients With Psychiatric Disorders, 2020
Source: JAMA Netw Open. 2021 Nov 23;4(11):e2134969. doi: 10.1001/jamanetworkopen.2021.34969 (PMC8611476; doi:10.1001/jamanetworkopen.2021.34969)
Supplement: Supplement. — eTable. ICD-10 Codes Used to Define Each Cohort and Logic Steps eFigure. Flowchart [file jamanetwopen-e2134969-s001.pdf]

Supplemental Online Content

Teixeira AL, Krause TM, Ghosh L, et al. Analysis of COVID-19 infection and mortality among patients with psychiatric disorders, 2020. *JAMA Netw Open.* 2021;4(11):e2134969. doi:10.1001/jamanetworkopen.2021.34969

eTable. ICD-10 Codes Used to Define Each Cohort and Logic Steps

eFigure. Flowchart

This supplemental material has been provided by the authors to give readers additional information about their work.

© 2021 Teixeira AL et al. *JAMA Network Open.*

<u>eTable:</u> ICD-10 Codes Used to Define Each Cohort and logic steps (#).

**Group 1: Schizophrenia spectrum**	**Group 2: Mood disorders**	**Group 3: Anxiety disorders**	**Group 4: Drug indicator only**	**Reference Group Exclusions**
**ICD-10 CODES**	**ICD-10 CODES**	**ICD-10 CODES**	**DRUG_CLASS**	Exclusion diagnoses used for Groups 1, 2, 3, 4 and the following
F20* (schizophrenia)	F30* (bipolar disorder)	F40* (phobic anxiety disorders)	Antipsychotics	F42* (Obsessive-compulsive disorder)
F22* (delusional disorders)	F31* (bipolar disorder)	F41* (other anxiety disorders)	Antidepressants; miscellaneous	F43* (Reaction to severe stress, and adjustment disorders)
F23* (acute and transient psychotic disorders)	F32* (major depressive disorder)		Selective serotonin reuptake inhibitor (SSRI) antidepressants	F44* (Dissociative and conversion disorders)
F25* (schizoaffective disorder)	F33* (major depressive disorder)		Serotonin and norepinephrine reuptake inhibitors	F45* (Somatoform disorders)
F28 (other nonorganic psychotic disorders)	F34* (persistent mood disorder)			F48* (Other nonpsychotic mental disorders)
F29 (unspecified nonorganic psychosis)	F39* (unspecified mood disorder)			F50* (Eating disorders)
**ICD 9 CODES**	**ICD 9 CODES**	**ICD 9 CODES**		F51* (Sleep disorders not due to a substance or known physiological condition)
295x	296x	300x		F53* (Mental and behavioral disorders associated with the puerperium)

© 2021 Teixeira AL et al. *JAMA Network Open.*

297x				F60* (Other specific personality disorders)
298x				F68* (Other disorders of adult personality and behavior)
**SNOMED CODE**	**SNOMED CODE**	**SNOMED CODE**		F69* (Unspecified disorder of adult personality and Behavior)
58214004	46206005	197480006		F90* (Attention-deficit hyperactivity disorders)
69322001				F95* (Tic disorder)
				F99 (Mental disorder, not otherwise specified)

(#) Logic Steps:
1. Use only people with a 2020 COVID-19 Test and a definitive Test Result;
2. Identify 'Psychiatry Diagnosis Group' by searching in data prior to 3/1/2020 for any of the diagnoses in table (in any coding position) and place them in category 1, 2, or 3 (If a person meets 2 or more groups, assign only to one group where 1 trumps 2 trumps 3);
3. Identify Group 4 by searching in patient reported medications AND/OR Prescriptions Written for DRUG_CLASS and select persons who did NOT fall into Group 1, 2 or 3: (these are people identified ONLY by drug and not by diagnosis code)
4. Identify Reference Group from remaining persons with a 2020 COVID-19 Test and a definitive Test Result, excluding those that meet exclusion criteria in Table.

© 2021 Teixeira AL et al. *JAMA Network Open.*

<u>eFigure:</u> Flowchart.

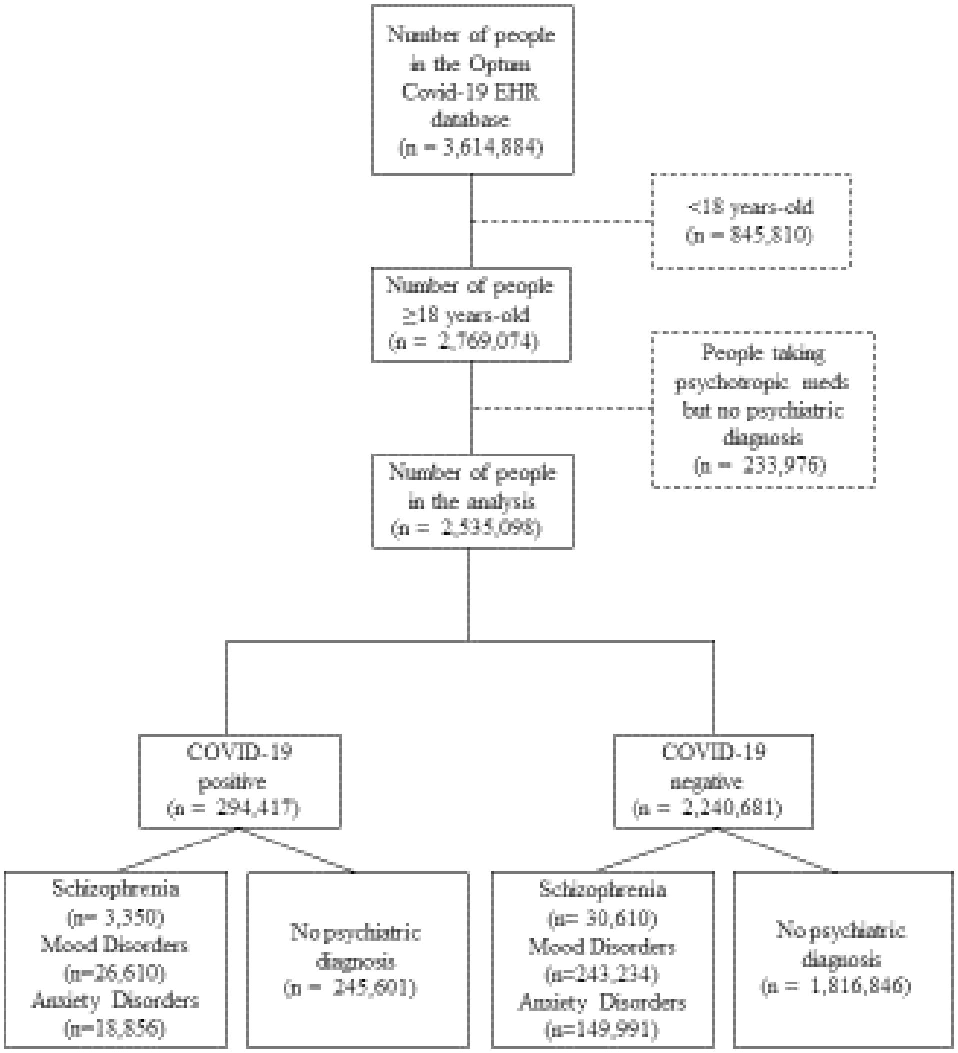

© 2021 Teixeira AL et al. *JAMA Network Open.*
